# Chronic Marijuana Use and Coronary Artery Disease: A Case Report of Two Patients

**DOI:** 10.7759/cureus.103726

**Published:** 2026-02-16

**Authors:** Resha R Ganthan, Jennifer Musumeci, Asher Gorantla, Francesco Rotatori

**Affiliations:** 1 Internal Medicine, Richmond University Medical Center, Staten Island, USA; 2 Cardiology, State University of New York (SUNY) Downstate Health Sciences University, Brooklyn, USA; 3 Cardiovascular Medicine, Richmond University Medical Center, Staten Island, USA

**Keywords:** cannabis (marijuana), cardiovascular risk factor, carotid atherosclerosis, coronary artery disease (cad), marijuana use, multivessel coronary artery disease (mvcad)

## Abstract

Cannabis is increasingly used worldwide, yet its cardiovascular effects remain incompletely understood. Emerging evidence suggests that chronic cannabis use may contribute to coronary artery disease (CAD) through hemodynamic stress, endothelial dysfunction, and proinflammatory pathways. We present a report of two patients with significant CAD and long-term daily cannabis use, highlighting its potential role as a modifiable cardiovascular risk factor. We describe two patients with long-term daily cannabis use and significant CAD. The first was a 34-year-old man with minimal traditional cardiovascular risk factors who presented with acute chest pain and was found to have single-vessel disease with an 80% stenosis of the posterior left ventricular branch requiring percutaneous coronary intervention. The second was a 52-year-old man with hypertension, hyperlipidemia, and obesity who presented with exertional chest pain and was found to have severe multivessel disease requiring referral for coronary artery bypass grafting. Both patients reported daily cannabis use for over 17 years. The first case demonstrates significant CAD in a young adult with few conventional risk factors, suggesting cannabis as a potential contributing factor. The second case illustrates severe multivessel disease in a patient with traditional risk factors, where long-term cannabis exposure may have accelerated disease progression. These cases align with current epidemiologic and mechanistic evidence linking chronic cannabis use to endothelial dysfunction, oxidative stress, and atherogenesis. Clinicians should inquire about cannabis use during cardiovascular risk assessments and consider its potential contribution to CAD, particularly in younger adults or in patients with unexpectedly severe disease. While causality cannot be definitively established from case reports, these cases support the need for further research into the long-term cardiovascular effects of chronic cannabis use.

## Introduction

Cannabis is one of the most commonly used psychoactive substances worldwide, with increasing prevalence due to legalization and shifting societal perceptions of safety [1,2]. While traditionally considered benign in otherwise healthy adults, emerging evidence suggests that chronic cannabis use may have significant cardiovascular effects, including arrhythmias, myocardial infarction, and coronary artery disease (CAD) [1-5]. The pathophysiology is multifactorial, involving acute hemodynamic stress, proinflammatory effects, endothelial dysfunction, and oxidative stress mediated by cannabinoid receptor signaling [6-11].

Despite these observations, cannabis is often underrecognized as a potential contributor to CAD, particularly in younger adults or those without conventional cardiovascular risk factors. We present a case series of two patients with significant CAD in the setting of long-term daily cannabis use: one young adult with minimal traditional risk factors and another middle-aged adult with established hypertension and hyperlipidemia. These cases highlight the potential role of cannabis as a modifiable risk factor in the development and progression of CAD and underscore the need for clinicians to assess cannabis exposure during cardiovascular risk evaluations [12].

## Case presentation

Case 1

History of Presentation

A 34-year-old African-American man with a history of childhood asthma and a 17-year history of daily marijuana smoking presented with acute chest pain that awakened him from sleep. The pain was substernal, nonradiating, and described as sharp and tight in quality, associated with nausea and diaphoresis. He reported smoking a marijuana cigarette approximately three hours prior to symptom onset. He denied a history of hypertension, diabetes mellitus, hyperlipidemia, or tobacco use. He also denied a family history of premature CAD.

Emergency medical services administered 325 mg of aspirin en route to the hospital. On arrival to the emergency department, his blood pressure was 118/83 mmHg, heart rate 68 beats/minute, respiratory rate 18 breaths/minute, oxygen saturation 98% on room air, and temperature 97.5°F. Given the concern for acute coronary syndrome based on typical chest pain and elevated troponin levels, he was treated as a presumed non-ST-segment elevation myocardial infarction and subsequently given a loading dose of clopidogrel 300 mg.

Investigations

**Figure 1:**
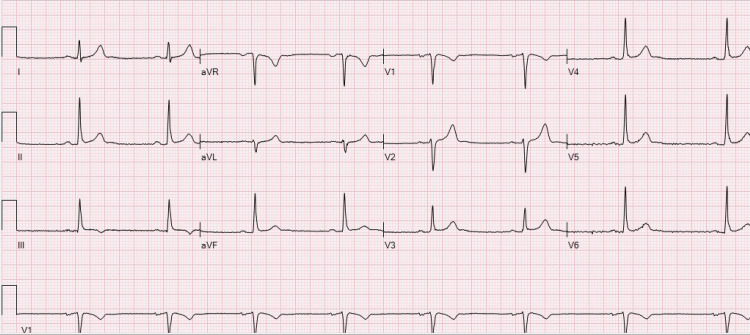
Case 1: electrocardiogram depicting sinus bradycardia with no significant ST-segment abnormalities aVR: augmented vector right; aVL: augmented vector left; aVF: augmented vector foot

An initial 12-lead electrocardiogram demonstrated sinus bradycardia at 48 beats/minute without ST-segment abnormalities (Figure 1).

Complete blood count and comprehensive metabolic panel were within normal limits. The initial high-sensitivity troponin was elevated at 156.9 ng/L with a rising trend on serial measurements. The lipid panel was within normal limits, and HbA1c was 6.9%. A summary of key laboratory values for both cases is shown in Table 1.

**Table 1:** Laboratory values for cases 1 and 2 WBC: white blood cell; LDL: low-density lipoprotein; TSH: thyroid-stimulating hormone

Laboratory test	Case 1	Case 2	Reference range
Hemoglobin (g/dL)	Normal	Normal	13-17
WBC (×10^9^°L)	Normal	Normal	4-11
Platelets (×10%L)	Normal	Normal	150-400
Creatinine (mg/dL)	Normal	Normal	0.6-1.3
Troponin (ng/L)	156.9 (initial, rising)	45 (peaked, lateralized)	<0.04
HbAlc (%)	6.9	5.4	<5.7
LDL (mg/dL)	90	Normal	<100
TSH (mIU/L)	0.381	Not reported	0.4-4.0
Free T4 (ng/dL)	1.2	Not reported	0.8-1.8
Urine toxicology	Positive for cannabinoids and opioids	Positive for cannabinoids	Negative

Thyroid studies showed mildly suppressed thyroid-stimulating hormone at 0.381 mIU/L, with a normal free T4 of 1.2 ng/dL. Urine toxicology was positive for cannabinoids and opioids; the opioid positivity was attributed to morphine administered in the emergency department. Transthoracic echocardiography demonstrated a preserved left ventricular ejection fraction (LVEF) of approximately 60%, mild left atrial dilation, normal right ventricular systolic function, with no notable valvular abnormalities.

**Figure 2:**
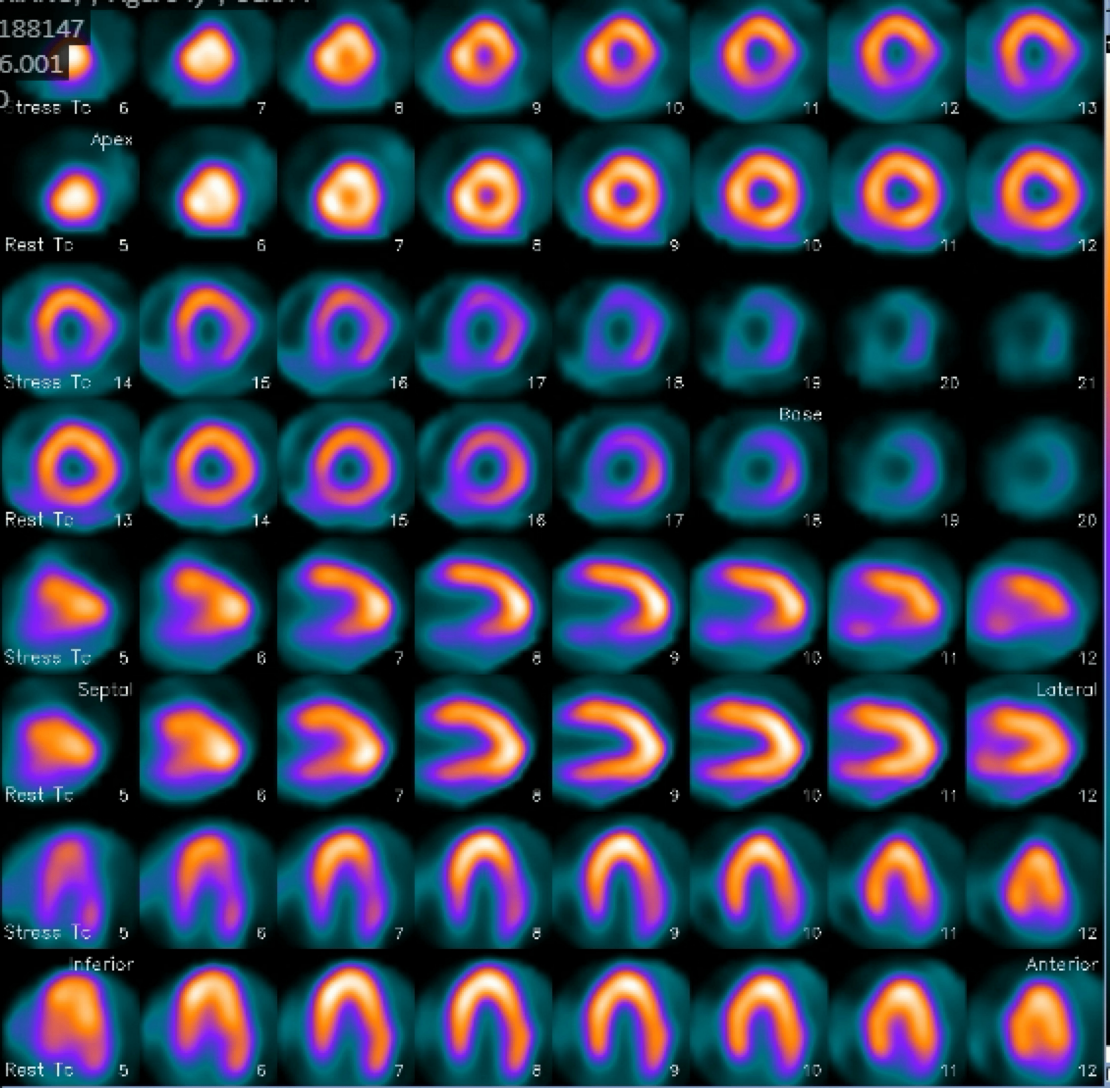
Case 1: abnormal exercise stress myocardial perfusion imaging study with large size, mild intensity, inferolateral wall reversible defect

**Figure 3:**
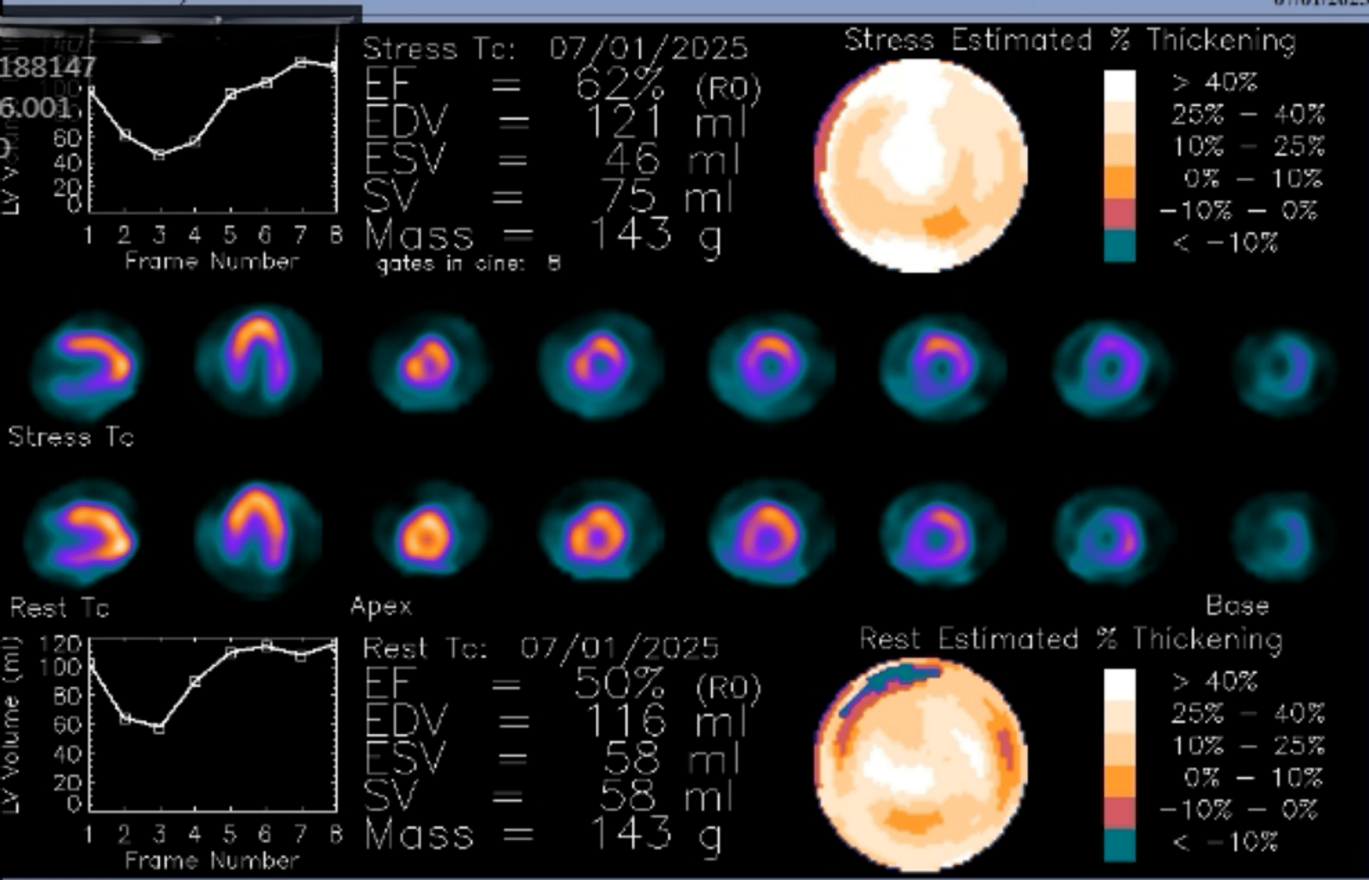
Case 1: left ventricular ejection fraction was estimated at 62% at rest and 50% with stress EF: ejection fraction; EDV: end-diastolic volume; ESV: end-systolic volume; SV: stroke volume

The patient remained chest pain-free with hemodynamic stability and no dynamic electrocardiographic changes, and was therefore scheduled for a nuclear stress test the following day for further ischemic risk stratification. A nuclear exercise myocardial perfusion study revealed a large, mild-intensity reversible defect in the inferolateral wall. LVEF was estimated at 62% at rest and 50% under stress, as shown in Figures 2, 3.

**Figure 4:**
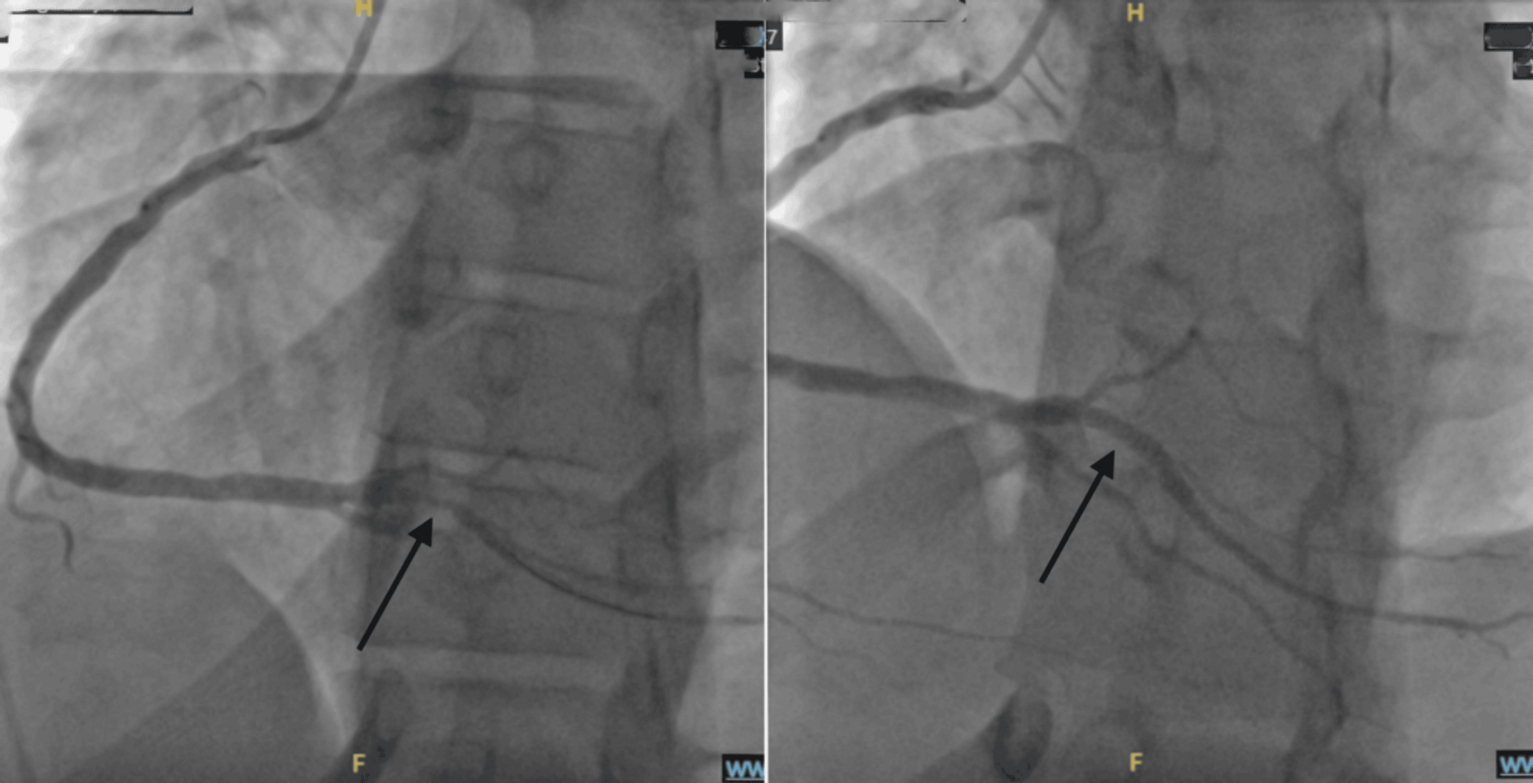
Case 1: left anterior oblique cranial views demonstrating a severe focal stenosis of the posterior left ventricular branch (left) with successful restoration of luminal caliber after percutaneous coronary intervention and stent placement (right)

Coronary angiography showed a large, patent left main coronary artery. The left anterior descending (LAD), left circumflex, and obtuse marginal (OM) arteries had only mild luminal irregularities. The right coronary artery was large and right-dominant with mild luminal irregularities. The posterior descending artery was patent. A large posterior left ventricular (RPLV) branch demonstrated an approximately 80% hazy tubular stenosis, as depicted in Figure 4. The patient underwent successful percutaneous coronary intervention with placement of a drug-eluting stent to the RPLV lesion.

Management

The patient was diagnosed with single-vessel CAD, with the 80% RPLV stenosis considered the culprit lesion. He was started on dual antiplatelet therapy with aspirin 81 mg daily and clopidogrel 75 mg daily, along with atorvastatin 80 mg daily. A beta-blocker was deferred due to baseline bradycardia. He was scheduled for an outpatient cardiology follow-up in four weeks and referred to cardiac rehabilitation. He was strongly counseled on cessation of marijuana use.

Case 2

History of Presentation

A 52-year-old man with a history of hypertension, hyperlipidemia, and more than 20 years of daily marijuana smoking presented with exertional chest pressure of one day’s duration. The initial episode occurred while shoveling snow and was relieved with rest. He later experienced recurrent chest discomfort while smoking marijuana, prompting presentation to the emergency department. His body mass index was 36 kg/m². He denied a family history of premature CAD. He was otherwise a nonsmoker and had no history of diabetes mellitus.

Investigations

A 12-lead electrocardiogram demonstrated a normal sinus rhythm at 71 beats/minute without ST-segment abnormalities. Initial high-sensitivity troponin levels were mildly elevated, peaking at 45 ng/L, with lateralization. HbA1c was 5.4%, and a lipid panel was within normal limits. Despite only mildly elevated biomarkers, the patient’s persistent symptoms and high clinical suspicion for CAD prompted coronary angiography. Transthoracic echocardiography revealed normal global systolic function with an LVEF of 60%-65% and normal valve function.

**Figure 5:**
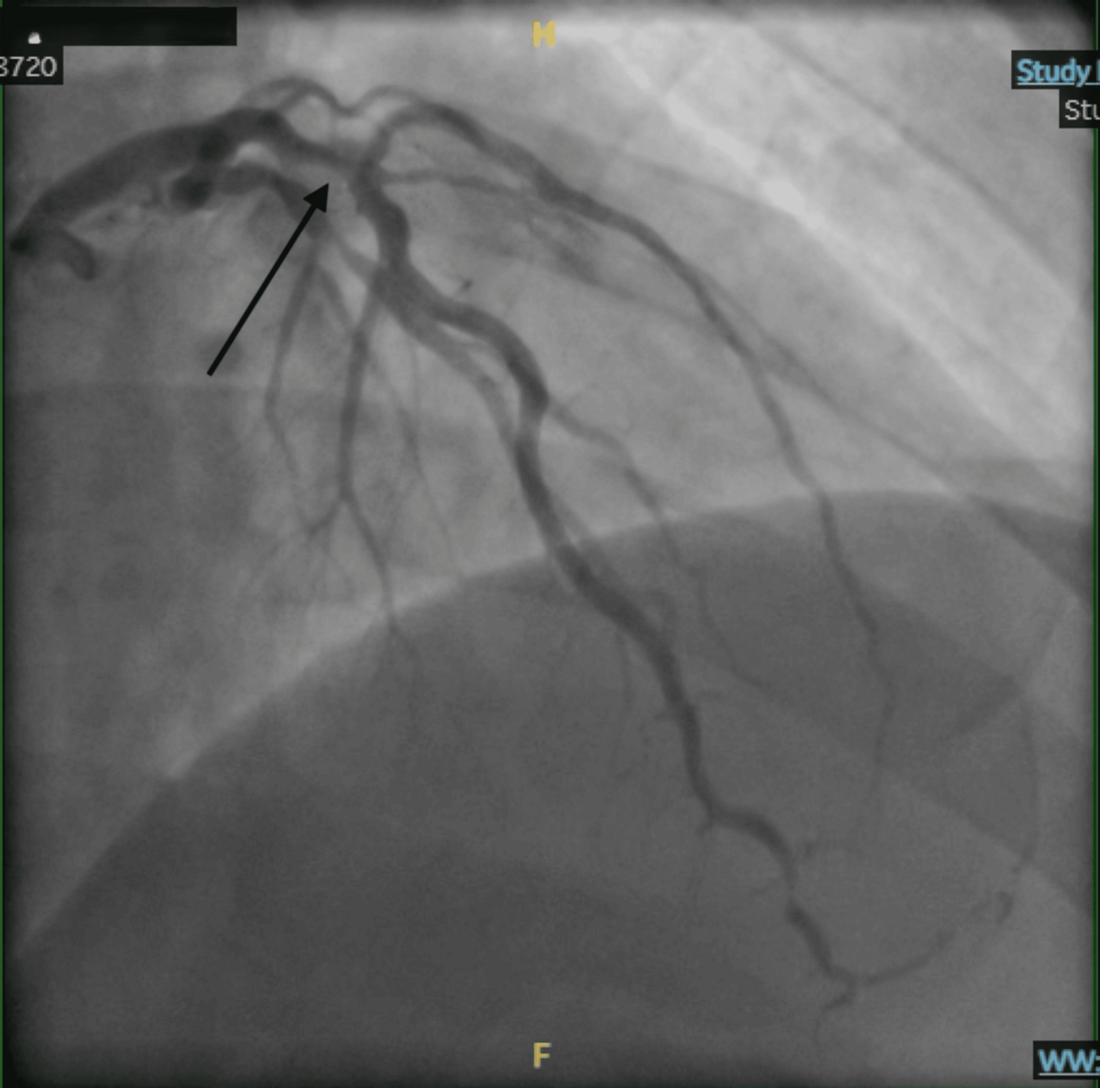
Case 2: right anterior oblique cranial view demonstrating severe stenosis of the proximal left anterior descending artery at the bifurcation into two large diagonal branches

Coronary angiography demonstrated significant multivessel disease. The proximal LAD artery at the bifurcation into two large diagonal branches exhibited approximately 80% stenosis, as shown in Figure 5.

The upper diagonal branch had 60%-70% disease, while the lower diagonal branch was occluded after its origin, with distal collateral filling. The OM1 branch was large, with a proximal lesion of 60% and distal occlusion of 80%. All other vessels demonstrated only mild, diffuse luminal irregularities. Based on the extent of disease, the patient was deemed a candidate for coronary artery bypass grafting (CABG).

Management

Given multivessel CAD, the patient was referred for surgical revascularization with CABG. He was discharged on aspirin 81 mg daily, atorvastatin 80 mg daily, and metoprolol succinate 25 mg daily. He was also counseled on cessation of marijuana use.

## Discussion

CAD remains a leading cause of morbidity and mortality worldwide. Traditional risk factors, such as hypertension, hyperlipidemia, diabetes, tobacco use, and family history, are well established, but the cardiovascular effects of chronic cannabis use are increasingly recognized given its rising prevalence [1-3].

Cannabis exerts complex hemodynamic and pathophysiologic effects. Acute use can increase heart rate, blood pressure, and sympathetic tone, potentially precipitating ischemia [1,4,5]. Chronic exposure may promote endothelial dysfunction and proatherogenic changes, as observed in long-term cannabis smokers and THC edible users [4,6]. Cannabinoid receptor signaling, particularly via CB1, may increase oxidative stress and inflammation, while CB2 may exert protective effects, indicating a nuanced role in atherosclerosis [10,11].

Epidemiologic evidence links frequent cannabis use with adverse cardiovascular outcomes. Daily users have higher odds of coronary heart disease, myocardial infarction, and stroke, even after adjusting for traditional risk factors [2]. Systematic reviews support these findings, particularly in younger adults and heavy users [3,5,6]. Case reports have also documented acute coronary syndromes, including vasospasm, in the setting of cannabis use [7,8].

The two cases illustrate possible clinical manifestations of chronic cannabis exposure. Case 1 shows significant CAD in a young adult with minimal traditional risk factors, suggesting cannabis as a potential contributor. Case 2 demonstrates severe multivessel disease in a patient with established risk factors, where chronic cannabis use may have accelerated disease progression [2,3,5].

This pattern suggests cannabis may act as a prominent risk factor in low-risk individuals and may exacerbate disease in patients with traditional risk factors. While causality cannot be definitively established, these cases highlight the importance of assessing cannabis use during cardiovascular risk evaluation and considering cessation counseling as part of risk modification [1,2,7]. Given the increasing prevalence of cannabis use worldwide, these cases also underscore the need for clinicians to routinely inquire about cannabis exposure as part of cardiovascular risk assessment and public health counseling.

It is important to emphasize that association does not equal causation. Confounding factors, variable exposure patterns, and limitations in observational data preclude definitive conclusions regarding a causal relationship between cannabis use and CAD [5,6]. Additionally, contemporary American College of Cardiology/American Heart Association guidelines highlight the value of intracoronary imaging modalities such as optical coherence tomography for risk stratification and procedural guidance in acute coronary syndromes, particularly in patients with complex or high-risk coronary lesions [13]. Nonetheless, these cases highlight the importance of inquiring about cannabis use during cardiovascular risk assessment, particularly in younger adults or those with unexpectedly severe disease, and suggest that counseling regarding cannabis cessation may be warranted as part of risk modification [1,2,7].

## Conclusions

This case series illustrates the potential contribution of chronic cannabis use to CAD. In the first patient, severe CAD developed in the absence of most traditional risk factors, although a mildly elevated HbA1c was present, suggesting that cannabis may have been a prominent contributor. In the second patient, existing cardiovascular risk factors were present, but long-term daily cannabis use may have accelerated the severity of multivessel disease. These observations are consistent with emerging epidemiologic and mechanistic evidence linking cannabis exposure to endothelial dysfunction, oxidative stress, and atherogenesis. While causality cannot be definitively established from case reports, clinicians should consider chronic cannabis use as part of cardiovascular risk assessment, particularly in younger adults or those presenting with disease disproportionate to conventional risk factors. For the purposes of this report, chronic cannabis use is defined as daily or near-daily consumption over multiple years. Cannabis use disorder is a complex condition involving a problematic pattern of cannabis use, ranging from mild to severe (addiction). This report is limited by the lack of precise quantification of cannabis dose and duration beyond patient self-report, as well as the potential for residual confounding from unmeasured risk factors. Further prospective research is warranted to clarify the impact of cannabis on CAD and to inform clinical guidelines for counseling and risk modification.
